# Time course analysis of gene expression identifies multiple genes with differential expression in patients with in-stent restenosis

**DOI:** 10.1186/1755-8794-4-20

**Published:** 2011-02-28

**Authors:** Santhi K Ganesh, Jungnam Joo, Kimberly Skelding, Laxmi Mehta, Gang Zheng, Kathleen O'Neill, Eric M Billings, Anna Helgadottir, Karl Andersen, Gudmundur Thorgeirsson, Thorarinn Gudnason, Nancy L Geller, Robert D Simari, David R Holmes, William W O'Neill, Elizabeth G Nabel

**Affiliations:** 1 National Heart, Lung, and Blood Institute (NHLBI), Division of Intramural Research, Bethesda, Maryland, USA; 2 Office of Biostatistics Research, National Heart Lung and Blood Institute, National Institutes of Health, USA; 3 Geisinger Clinic, Danville, Pennsylvania, USA; 4 Division of Cardiovascular Medicine, Ohio State University Medical Center, Columbus, Ohio, USA; 5 Department of Cardiovascular Medicine, Wellcome Trust Centre of Human Genetics, Oxford, UK; 6 Cardiovascular Research Center, Landspitali University Hospital, Reykjavik, Iceland; 7 University of Iceland, Reykjavik, Iceland; 8 Divsion of Cardiovascular Diseases, Mayo Clinic, Rochester, Minnesota, USA; 9 Division of Cardiology, Department of Internal Medicine, Miller School of Medicine, University of Miami, Miami, Florida, USA

## Abstract

**Background:**

The vascular disease in-stent restenosis (ISR) is characterized by formation of neointima and adverse inward remodeling of the artery after injury by coronary stent implantation. We hypothesized that the analysis of gene expression in peripheral blood mononuclear cells (PBMCs) would demonstrate differences in transcript expression between individuals who develop ISR and those who do not.

**Methods and Results:**

We determined and investigated PBMC gene expression of 358 patients undergoing an index procedure to treat in de novo coronary artery lesions with bare metallic stents, using a novel time-varying intercept model to optimally assess the time course of gene expression across a time course of blood samples. Validation analyses were conducted in an independent sample of 97 patients with similar time-course blood sampling and gene expression data. We identified 47 probesets with differential expression, of which 36 were validated upon independent replication testing. The genes identified have varied functions, including some related to cellular growth and metabolism, such as the *NAB2 *and *LAMP *genes.

**Conclusions:**

In a study of patients undergoing bare metallic stent implantation, we have identified and replicated differential gene expression in peripheral blood mononuclear cells, studied across a time series of blood samples. The genes identified suggest alterations in cellular growth and metabolism pathways, and these results provide the basis for further specific functional hypothesis generation and testing of the mechanisms of ISR.

## Background

Cardiovascular disease is the leading cause of death in western countries and a major cause of morbidity and mortality world-wide. Studying coronary atherosclerotic disease (CAD) is challenging for several reasons, since it has substantial environmental and genetic components. Furthermore, despite the nearly universal presence of coronary atherosclerosis, particularly as individuals age, cardiovascular events such as acute coronary syndromes, sudden death and the need for revascularization therapy only occur in some individuals, highlighting the difficulty in precisely defining atherosclerosis phenotypes. In symptomatic patients, revascularization therapy is often required, and percutaneous intervention with balloon angioplasty and stent implantation is a cornerstone of therapy. In-stent restenosis (ISR) is a late complication of stent implantation in which inflammatory and proliferative responses to the vascular injury caused by angioplasty and stenting lead to neointimal hyperplasia within the stent and at its edges over the following weeks and months. Many of the same inflammatory and proliferative processes are activated in the development of atherosclerosis but occur over years or decades. ISR is characterized by proliferative responses to the vascular wound incurred as a result of stent implantation[1]. Therefore, ISR may be viewed as a model phenotype of vascular wound repair for which the mechanisms represent part of the pathologic picture of atherosclerosis, with relatively accelerated wound repair responses operative in the vascular wall and in peripheral blood leukocytes.

In the analysis reported here, we apply a novel method to analyze time-course gene expression data to gene expression profiles of peripheral blood mononuclear cells (PBMCs) of patients enrolled in our study of ISR. The results of the discovery transcriptome analysis of the CardioGene Study were tested for replication in an independent sample of Icelandic patients. We ultimately identified and validated a set of 32 genes that are temporally differentially expressed in the blood of patients who develop ISR after stenting, compared to those who do not develop ISR, highlighting the importance of cellular growth pathways and identifying several biologic candidates for further mechanistic investigation.

## Results

### Microarray data quality control and filtering

**Figure 1:**
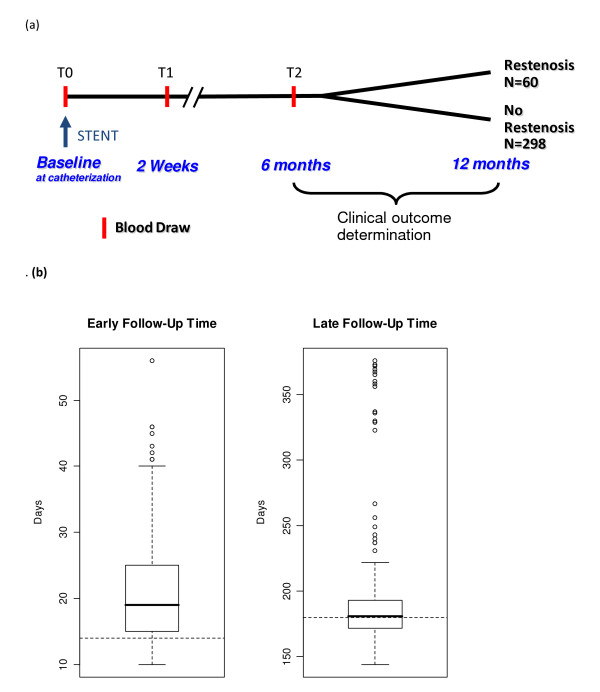
**The CardioGene Study Design and intervals for blood sampling**. (a) Times of blood sampling and clinical follow-up. Blood draws occurred at times T0 (pre-stent), T1 (2-4 weeks post-stent) and T2 (six months post-stent) (b) Distribution of the intervals for blood sampling from baseline to early follow-up (2-4 weeks post-stent) and late follow-up (6-12 months) time points, are shown for the CardioGene Study.

In the CardioGene Study, 312 patients were included (52 with ISR, and 260 who did not develop ISR, as defined by criteria for clinical restenosis[2]) after quality control filtering (Table 1). All 312 patients had baseline gene expression profile data (Figure 1a). Of these, 203 had a blood sample and high quality gene expression profile data at early follow-up (2-4 weeks post-stent), and 166 had high quality gene expression profile data at both early follow-up and late follow-up (6 months post-stent). A total of 681 samples were included in the time course analysis. Box plots showing the distributions of the early and late follow-up times are presented in Figure 1b. From the deCODE sample of patients in Iceland, 97 patients were enrolled and had high quality gene expression profiles at baseline (28 with ISR, 69 without ISR). Of these, 86 patients had a follow-up blood sample and acceptable gene expression data at 6 months post-stent. Thus, a total of 183 samples were included in the replication analysis.

**Table 1:** Clinical characteristics of the CardioGene and deCODE cohorts

	CardioGene N = 312	deCODE N = 97
Number of female (% female)	100 (32.0%)	19 (19.6%)
Age (mean ± sd)	65.6 (10.4)	64.8 +/- 10.0
Diabetes	91 (29.2%)	12 (12.5%)
Hypertension	213 (68.2)	58 (64%)
Ever smoked	194 (62.1%)	23 (48%)
Hyperlipidemia	241 (77.2%)	47 (48.4%)
Prior ISR (N, %)	10 (4.3%)	----
ISR	52 (16.7%)	28 (28.9%)
Acute coronary syndrome at time of index PCI	64 (20.5%)	----
Reference diameter (mean ± sd)	2.74 mm +/- 0.74 mm	2.71 mm +/- 0.050 mm
Lesion length (mean ± sd)	9.4 mm +/- 5.3 mm	12.7 mm +/- 8.14 mm
LAD location (% with stent in LAD)	126 (40.6%)	52 (53.6%)
Post-stent % stenosis in patients with ISR	10.96 mm +/- 6.2 mm	6.0 mm +/- 12.7 mm
Number of timepoints at which PBMC gene expression measured	3	2

### CardioGene discovery analysis of time-course RNA expression in PBMCs

**Figure 2:**
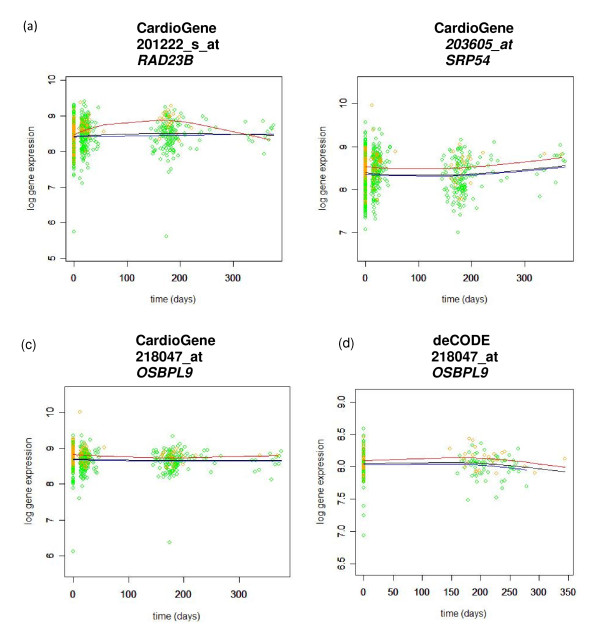
**Patterns of time course results for three probe sets in the CardioGene and deCODE studies**. The color of the lines plotted is red for ISR, blue for non-ISR, and black for the model fitted to all patients, under the null hypothesis. Probeset 201222_s_at (a) shows a divergent pattern of gene expression between individuals who develop ISR and those who do not, whereas 203605_at (b) shows consistently differential expression across the timecourse. Gene expression for probe set 218047_at shows similar gene expression patterns in both the CardioGene (c) and deCODE (d) cohorts.

Applying the time varying intercept model, 46 probes, corresponding to 42 distinct genes, were found to be significant at FDR adjusted q-value less than 0.05 out of 12467 probe sets that passed the quality control (Additional File 1). Two patterns of gene expression were observed, with some genes showing divergent gene expression patterns across the time course (Figure 2a) and others showing consistently differential gene expression over the time course (Figure 2b). Descriptions of the genes to which these probe sets map are provided in Additional File 1.

### Replication analysis using deCODE time course samples

**Figure 3:**
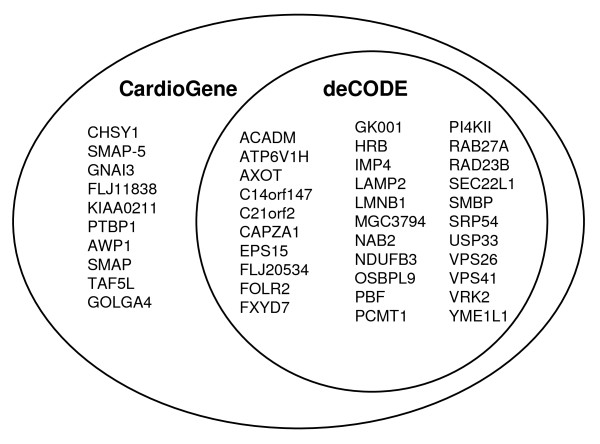
**Genes identified in the CardioGene discovery analysis and replicated in the deCODE analysis**. A Venn diagram shows the genes identified in the CardioGene discovery analysis and those replicated in the deCODE analysis.

Among 46 significant probe sets identified in the CardioGene time-course analysis, 36 probe sets had FDR adjusted q-value less than 0.05 in the deCODE replication analysis (Figure 3). Gene expression patterns were largely consistent with the patterns observed in the CardioGene discovery analysis (Figure 2c and 2d). The 36 probe sets mapped to 32 unique genes (Table 2).

**Table 2:** Validated genes x(32 genes, 36 probesets) with annotation information and gene expression patterns, as shown in Figure 2

Affymetrix probesets	Gene Symbol	Gene Name	Gene expression pattern
202502_at	ACADM	acyl-Coenzyme A dehydrogenase, C-4 to C-12 straight chain	Consistent (ISR > No-ISR)

221504_s_at	ATP6V1H	ATPase, H+ transporting, lysosomal 50/57 kDa, V1 subunit H	Consistent (ISR > No-ISR)

202654_x_at	AXOT	axotrophin	Consistent (ISR > No-ISR)

212460_at	C14orf147	chromosome 14 open reading frame 147	Divergent (ISR > No-ISR)

203994_s_at	C21orf2	chromosome 21 open reading frame 2	Consistent (No-ISR > ISR)

203996_s_at	C21orf2	chromosome 21 open reading frame 2	Consistent (No-ISR > ISR)

208374_s_at	CAPZA1	capping protein (actin filament) muscle Z-line, alpha 1	Consistent (ISR > No-ISR)

217886_at	EPS15	epidermal growth factor receptor pathway substrate 15	Consistent (ISR > No-ISR)

218646_at	FLJ20534	hypothetical protein FLJ20534	Consistent (ISR > No-ISR)

204829_s_at	FOLR2	folate receptor 2 (fetal)	Consistent (ISR > No-ISR)

220131_at	FXYD7	FXYD domain containing ion transport regulator 7	Consistent (No-ISR > ISR)

217814_at	GK001	GK001 protein	Consistent (ISR > No-ISR)

218092_s_at	HRB	HIV-1 Rev binding protein	Consistent (ISR > No-ISR)

212411_at	IMP4	U3 snoRNP protein 4 homolog	Divergent (No-ISR > ISR)

200821_at	LAMP2	lysosomal-associated membrane protein 2	Consistent (ISR > No-ISR)

203276_at	LMNB1	lamin B1	Consistent (ISR > No-ISR)

214773_x_at	MGC3794	putative MAPK activating protein	Consistent (ISR > No-ISR)

212803_at	NAB2	NGFI-A binding protein 2 (EGR1 binding protein 2)	Consistent (No-ISR > ISR)

203371_s_at	NDUFB3	NADH dehydrogenase (ubiquinone) 1 beta subcomplex, 3, 12 kDa	Consistent (ISR > No-ISR)

218047_at	OSBPL9	oxysterol binding protein-like 9	Consistent (ISR > No-ISR)

221123_x_at	PBF	papillomavirus regulatory factor PRF-1	Consistent (No-ISR > ISR)

208857_s_at	PCMT1	protein-L-isoaspartate (D-aspartate) O-methyltransferase	Consistent (ISR > No-ISR)

205202_at	PCMT1	protein-L-isoaspartate (D-aspartate) O-methyltransferase	Consistent (ISR > No-ISR)

209345_s_at	PI4KII	phosphatidylinositol 4-kinase type II	Consistent (No-ISR > ISR)

201222_s_at	RAD23B	RAD23 homolog B (S. cerevisiae)	Divergent (ISR > No-ISR)

201223_s_at	RAD23B	RAD23 homolog B (S. cerevisiae)	Consistent (ISR > No-ISR)

209207_s_at	SEC22L1	SEC22 vesicle trafficking protein-like 1 (S. cerevisiae)	Consistent (ISR > No-ISR)

217758_s_at	SMBP	SM-11044 binding protein	Consistent (ISR > No-ISR)

203605_at	SRP54	signal recognition particle 54 kDa	Consistent (ISR > No-ISR)

212513_s_at	USP33	ubiquitin specific protease 33	Consistent (ISR > No-ISR)

201807_at	VPS26	vacuolar protein sorting 26 (yeast)	Consistent (ISR > No-ISR)

210849_s_at	VPS41	vacuolar protein sorting 41 (yeast)	Divergent (ISR > No-ISR)

205126_at	VRK2	vaccinia related kinase 2	Divergent (ISR > No-ISR)

216304_x_at	YME1L1	YME1-like 1 (S. cerevisiae)	Consistent (ISR > No-ISR)

201351_s_at	YME1L1	YME1-like 1 (S. cerevisiae)	Consistent (ISR > No-ISR)

222294_s_at	RAB27A		Consistent (ISR > No-ISR)

### Annotation of Genes Identified

**Figure 4:**
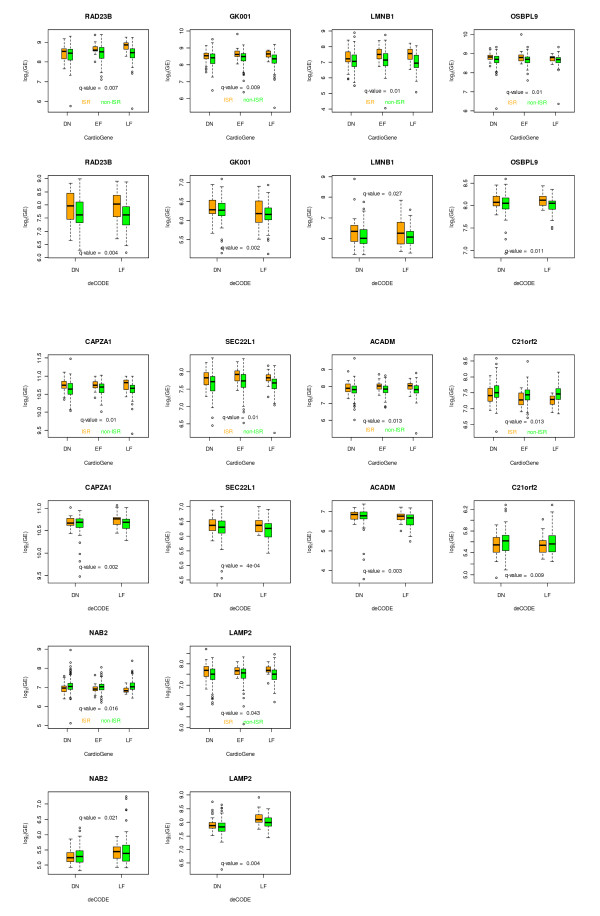
**Gene expression for the top eight genes, *NAB2 *and *LAMP2***. Gene expression plots are shown for the top eight ranked genes, and *NAB2 *and *LAMP2 *genes are shown together, as these genes have known functions relevant to ISR. Gene expression data is shown for both cohorts across the time course of samples in each study. For each gene, expression levels are shown on the y-axis as the log 2 of normalized gene expression (GE), and the timepoints shown on the x-axis of each plot correspond to baseline (BL), early follow-up (EF), and late follow-up (LF) timepoints, although data was analyzed across the continuous timeframe of follow-up blood sampling, rather than categorically as is depicted here for illustration purposes. The box plots are plotted with the whiskers at 1.5 times the interquartile range.

Using the DAVID/EASE annotation tool, several categories of genes were identified, falling into multiple gene ontology classifications. No single category achieved a significance score indicating statistically significant over-representation of a specific gene ontology, and several gene ontology classes showed multiple genes (Additional File 2). The categories with the highest representation (i.e. the largest number of genes mapping to these ontologies) included cell growth and/or maintenance, cellular metabolism, catalytic activity, nucleotide and nucleic acid metabolism, transport, protein binding and cellular binding. Genes with prior evidence of expression in the arterial wall after vascular injury included the *NAB2 *and *LAMP2 *genes, with data in the prior literature suggestive of functional relevance of these genes, by mediating proliferative responses and recruitment of PBMCs to injured endothelium, respectively[3-13] (Figure 4).

### Sensitivity Analysis

In a sensitivity analysis, we found that the median number of significant probe sets using the same criteria of FDR 0.05 was only 2, the mean was 3.04, and the maximum number was 14 in one iteration, demonstrating a significantly low likelihood that the probesets we identified and replicated were identified due to chance.

### Hypothesis-driven analysis of gene expression

We compared our results to published studies examining gene expression in ISR, chiefly in human vascular tissue samples [14,15]. In one study, peripheral blood total leukocytes were also examined [14]. In the CardioGene baseline blood sample, genes reported by Ashley et al were examined [15], and none were significantly differentially expressed in the baseline blood sample or in our timecourse results. In another study by Zohlnhofer et al [14], gene expression data from neointimal vascular tissue and peripheral blood leukocytes were reported. We examined those genes meeting a Bonferroni multiple testing correction from this discovery report and did not find overlap with our gene expression findings.

## Discussion

Using a repeated sampling study design, we investigated PBMC transcript expression in patients undergoing stent implantation, using a novel time-course analysis method. We identified a set of 42 genes with differential temporal expression among patients with and without ISR at one year follow-up in a discovery analysis of the CardioGene Study. Independent replication testing in an Icelandic sample confirmed differential expression of 36 probesets mapped to 32 genes. The gene expression patterns over time may be of interest as well, with consistently expressed genes representing gene expression data that may be able to predict ISR and differentially expressed genes over the time course representing genes with possible direct functional roles in the development of ISR, both of which require further investigation to explore more fully.

Gene expression profiling with DNA microarray technology is a popular tool to monitor the expression level of thousands of genes simultaneously and has been applied in cardiovascular research, to detect patterns of gene expression indicative of underlying disease states[16-21]. Since the data generated represent the temporal abundance of mRNA levels in the sample, measurements of the change of this abundance over the course of disease progression (or any biological process) is therefore both possible and of great scientific interest using this technology. In fact, Yuan and Kendziorski[22] reported that more than one-third of the experiments catalogued in the Gene Expression Omnibus (National Center for Biotechnology Information; http://www.ncbi.nlm.nih.gov/geo/) are from experiments that measure gene expression over time. Early time-course RNA expression studies have focused on identifying clusters of genes with a similar pattern over time[14,23,24]. More recently, detecting a differential gene expression pattern over time between several biological groups has become an interesting goal of time-course gene expression data.

To detect differential gene expression pattern over time, we used a time-varying intercept model which can account for differences in sample intervals between patients. The term "differential gene expression pattern over time" can be interpreted in several ways to form suitable questions and, thus, the hypotheses are dependent on the particular experiment under consideration. We considered two related questions where each can roughly correspond to the main effect of the group, and the interaction between group and time points. Consider, for example, a gene with exactly the same expression pattern over time in both groups, but the first group has a higher expression than the second group, consistently over all time points. This is a gene that shows the main effect of the group. On the other hand, consider another gene with similar expression level at two time points, 1 and 2, but this gene's expression increases from time 1 to 2 in one group but decreases in another group. This group-by-time interaction cannot be examined by methods that only test the main effect of the group. The time-varying intercept model we used can detect both the main effect and group-by-time interaction. This method, however, requires a large number of bootstrap resampling to evaluate the significance level of the difference between groups, which can be computationally challenging especially when a large number of genes are tested.

Of the genes we identified, the most extensive prior literature in vascular disease was found for the *NAB2 *gene, which is also known as EGR1 binding protein 2. Early growth response (EGR) genes, which are transcription factors that are implicated in a wide variety of proliferative and differentiative processes[15]. Nab proteins are necessary for Schwann cells to exit the cell cycle and generate a myelin-specific gene profile and are key regulators of the myelination process of peripheral nerves[25]. *NAB2 *is expressed in vascular smooth muscle cells in response to injury[3-12,26] and EGR1 has been identified in a microarray study of in vitro smooth muscle cell proliferation[27]. The *LAMP2 *gene product protects the lysosomal membrane from proteolytic enzymes within lysosomes and also functions as a receptor for proteins to be imported into lysosomes[28]. Mutations in the *LAMP2 *gene have been identified in patients with hypertrophic cardiomyopathy[29], and the gene product mediates adherence of PBMCs to the vascular endothelium[13]. Cellular adhesion to the vascular endothelium has been well-described in animal models and post-mortem human examinations, in atherogenesis and acute vascular injury[30,31]. In the latter, the extent of leukocyte adhesion is predictive of the degree of subsequent neointimal hyperplasia, which is the key lesion of ISR.

Some genes identified have no apparent role in vascular biology, such as *VPS26, VPS41*, *SRP54*, and *RAD23B*, and comparison to previously published reports in the literature do not show differential gene expression in other studies of restenosis, although these investigations were conducted primarily on vascular tissues or culture vascular smooth muscle cells rather than peripheral blood[17,18,27,32]. The *VPS26 and VPS4 *genes belongs to a group of vacuolar protein sorting genes and may have a role in lysosome maintenance[33], and *SRP54 *is a protein in the signal recognition particle, which directs secretory proteins to membranes as they emerge from the ribosome [34]. Specific vascular or inflammatory cell function has not been described, but derangement of these basic cellular processes may impact vascular and other physiologic functions adversely. *RAD23B *has a role in DNA (nucleotide excision) repair, and genetic variants in *RAD23B *have been associated with several solid tumors[35-38]. The association with cancer would suggest a possible link to excess proliferative mechanisms in vascular wound repair, as has been described for many other cell cycle regulatory genes[39]. Genetic variants in other genes we identified are also associated with human diseases. Several genetic variants in *ACADM *have been associated with medium-chain acyl-CoA dehydrogenase activity, but there is no known vascular implication of this disorder[40]. Variants in *PCMT1 *and *FOLR2 *have been associated with neural tube defects[41-43], with no known vascular phenotype in these cases. Overall the findings of this study are hypothesis-generating and can be used to support the rational for investigating the function of specific genes and pathways in adverse vascular remodeling, which is relevant to both ISR and more general CAD phenotypes.

We compared the results of our study to previously published reports of transcriptome analysis in ISR. Our results were negative for replication of these studies which focused primarily on vascular tissue samples, in relatively small sample sets. In one study, peripheral blood total leukocyte gene expression was studied in 10 patients with ISR and atherectomy specimens [23]. While a high degree of correlation between peripheral blood leukocyte and arterial neointima tissue gene expression was identified in a subset of genes, these findings were based upon single measurements in a small sample size and were not replicated in the original report. These prior reports highlight the major difficulty of studying vascular tissues, since access to these tissues in adequately large sample sizes is limited.

Vascular biopsy and atherectomy are performed infrequently as part of routine clinical care and would not support well-powered studies of vascular tissues. Tissue sampling over a time course is not clinically indicated or possible. Additionally, a large degree of intra-individual variability in gene expression was noted in these prior studies of vascular tissues, making replication testing critical, yet this cannot be done without access to additional tissues samples. In our study, we analyzed peripheral blood leukocytes, specifically focusing on the mononuclear fraction which contains primarily B and T lymphocytes and monocytes. Although the analysis of peripheral blood cells would ideally be complemented by similar studies in vascular tissues, studying gene expression profiles in blood leukocytes is biologically relevant due to well-defined interactions with the arterial wall, particularly in the setting of vascular injury and repair as in the setting of ISR[17]. The overlap between vascular and blood gene expression in one prior transcriptome analysis of ISR was supportive of our rationale to study PBMCs. For these reasons, and with the additional prior knowledge that inflammation plays a significant role in the development of ISR, we undertook a study of PBMCs in several hundred patients, with adequately powered replication testing for our top discovery findings. Additionally, we use a time course analysis method that improved our ability to detect gene expression signals between the two comparison groups, overcoming some of the difficulty of substantial variability in single point microarray gene expression data.

To address the possibility of false positives identified with our statistical methods, we conducted replication analyses in the independent sample of deCODE samples and we conducted bootstrap resampling to assess significance of the findings. Through this sensitivity analysis, we demonstrated that the validation of 36 probe sets is not likely to be due to chance. Additional potential limitations of this study of ISR are the use of a clinical restenosis outcome, rather than an angiographic outcome, in which clinically silent ISR may have been missed, and the choice of tissue analyzed, as discussed. The CardioGene and deCODE cohorts differ in the incidence of ISR (16.7% in the CardioGene Study and 28.8% in deCODE) with the patients in the deCODE sample showing overall lower residual percent stenosis in the treated lesion after stent implantation. Also, the proportions with hyperlipidemia and diabetes differ. However, despite the differences in the cohorts, we find replication of a substantial proportion of the discovery findings.

## Conclusions

In summary, we have used a method to analyze gene expression in serial blood samples and identified a set of genes that show differential expression in the blood of patients who develop ISR after BMS implantation, compared to those who do not. These gene expression patterns of adverse vascular remodeling suggest possible hypotheses for the mechanisms of injury-induced remodeling observed in both ISR and CAD, since ISR is a niche phenotype occurring in a subset of patients with CAD. Further studies are needed to investigate the functional relevance of these genes and are warranted based upon the findings of this study.

## Methods

### Study samples

The CardioGene Study was an IRB-approved, prospective cohort study of 358 patients enrolled at the time of bare metal stent (BMS) implantation to treat de novo, previously untreated native coronary artery lesions at William Beaumont Hospital (Royal Oak, Michigan, USA) and the Mayo Clinic (Rochester, Minnesota, USA). Patients were followed for one year to determine ISR outcomes (Figure 1). Enrollment began in February 2002 and was closed in September 2003, prior to the approval and clinical use of drug-eluting stents (DES) in the United States. The protocol was approved by the NHLBI IRB as well as the IRB at each of the clinical enrollment sites. Informed consent was provided by each patient. Standardized case report forms were used to collect baseline clinical data and outcome information in follow-up[2]. Since gene expression discovery studies may suffer from false positives, despite the use of statistical corrections for multiple hypothesis testing, we sought out independent validation of the primary findings of the CardioGene Study in a separate cohort. For independent replication testing, 97 patients undergoing stent implantation with BMS were enrolled in Iceland by collaborators at Landispitali University Hospital and University of Iceland, and RNA samples were provided through a contract with deCODE Genetics, Inc. The study was approved by the National Bioethics Committee and the Data Protection Authority of Iceland, and each patient provided informed consent for participation in the study. Clinical characteristics of both study cohorts are summarized in Table 1.

### Clinical phenotype

Consecutive patients presenting to the cardiac catheterization laboratories of the clinical enrollment sites were approached for participation in the study. Follow-up clinical evaluation was performed via patient interview and review of all available medical records at 6 months and 12 months post-stent (Figure 1). ISR was defined as clinical restenosis[2], which was defined by ischemic symptoms after stent implantation and evidence of flow limitation in the treated vessel by either invasive or non-invasive testing. Follow-up angiography was not specifically performed for the CardioGene Study. Any available angiographic data performed as part of each patient's clinical care was recorded. The Icelandic subset had follow-up angiography of all patients at 6 months. Quantitative coronary angiography (QCA) in the CardioGene Study was performed by a single QCA reader, and by a TIMI core QCA center for the Icelandic subset.

### PBMC Microarray Analysis

Whole blood was sampled immediately prior to stenting, 2-4 weeks after stenting and six months post-stent. Blood samples were collected into EDTA-containing tubes at three times from patients enrolled in the CardioGene Study (Figure 1), and two time from patients enrolled in Iceland. Samples from each time point were handled according to the standardized blood handling and RNA isolation protocols[2], for consistency across time points and clinical enrollment sites. PBMCs were harvested by technicians at each clinical enrollment site following standardized protocols for Ficoll separation of whole blood and osmostic lysis of red blood cells, to avoid technical variation and to minimize globin mRNA contamination. All blood processing was completed within 4 hours of blood draw, at room temperature. Snap-frozen PBMCs samples were shipped to the NIH laboratory, and RNA isolation was performed using the Qiagen RNA isolation protocol, with DNase treatment. RNA labeling and microarray image analysis was according to the Affymetrix U133A protocol. To minimize the impact of batch-to-batch variation, the multiple samples from each patient were processed together through all stages, and the order of RNA labeling and microarray hybridization was randomized. The same methods were used in the CardioGene and deCODE study samples, and microarray analysis was conducted on all samples at the NIH. In both studies, technicians performing the assays were blinded to the clinical status of patients.

### Statistical Analysis

Raw image data were normalized using RMA (Robust multichip average) normalization[44]. Several steps of quality control were performed. These included the investigation of the residual plots using affyPLM, gender match using a linear discriminant analysis and shrunken centroid method[45-47]. Probes with values less than 6 in log 2 scale in more than two thirds of samples were excluded, and age and gender were adjusted before the analysis[48].

In the study design of the time course of blood gene expression profiling, the follow-up times were set at 2 weeks and 6 months for the early and late follow-up. A window was set at each of the time points, with the early follow-up designated as any time 2-4 weeks post-stent and the late follow-up designated as any time between 5-7 months for patients seen at 6 months. If patients did not return for follow-up at the 6 month time point, an attempt was made to have the patient provide the late follow-up blood sample at 12 months post-stent, at the time when final clinical ascertainment for ISR was made. As a result of the use of time windows, we were able to increase ascertainment of follow-up blood samples, but the actual sample collection intervals after stent implantation were not precisely spaced. To analyze such data, we used the time varying intercept model using a B-spline basis of dimension 2 based on only one knot at the median follow-up time (which was 14 days) (Additional File 3)[49]. This is because the majority of the early follow-up is centered around 14 days. The model was fit under the null hypothesis using combined samples of ISR and non-ISR patients, and under the alternative hypothesis where separate models were fitted for ISR and non-ISR patients, respectively. The test statistic is the improvement in the fitted model under the alternative hypothesis from that under the null hypothesis, as measured by the relative difference in the residual sum of squares. The bootstrap method was used to estimate the p-value of this test statistic[47,49]. To obtain accuracy in estimating the p-values, 10 million bootstrap samples were generated. Significant probe sets were selected based on an FDR-adjusted q-value less than 0.05[50].

The replication analysis was performed in an independent set of samples, using deCODE samples from patients enrolled in Iceland, to validate probe sets identified by the time course analysis of the CardioGene samples. We performed the same analysis using the time varying intercept model. P-values were estimated based on 100,000 bootstrap samples, and FDR adjusted q-value of 0.05 was, again, used to select significant probe sets. Here the adjustment was based on the selected set of genes since it was a replication analysis. As a sensitivity analysis and to evaluate whether any random set of 46 probe sets would result in significant results as we saw in the analysis of deCODE samples, we randomly selected 46 probe sets, and repeated the validation analysis. This procedure was repeated 100 times, and the number of probe sets yielding an FDR value less than 0.05 was observed.

Data analyses were performed based on the R language utilizing libraries freely available via the Bioconductor project.

Box plots were generated using the default R software options. The upper whisker is the data point which is 1.5 times the difference between 75th percentile and median from the upper bound of the box, and lower whisker is 1.5 times the difference between median and 25th percentile from the lower bound of the box. Overall, the whisker represents the data point which is 1.5 times the interquartile range from the box.

Finally, to evaluate genes identified in a prior studies of ISR [23,24], we analyzed the reported genes in our data, at the baseline timepoint comparing normalized gene expression values between ISR cases and no-ISR controls and also against our timecourse analysis results.

### Pathway analysis

Annotation of probe sets was done using the NetAffx (Affymetrix U133A library set) and the DAVID/EASE software, which provides gene name and functional annotation of probe sets[51,52]. Analysis of over-representation, using a Fisher's exact test of over-representation of genes from different functional categories based on gene ontology classifications, was performed.

## List of abbreviations

BMS: bare metal stents; CAD: coronary atherosclerotic disease; DES: drug-eluting stents; ISR: in-stent restenosis; PBMC: peripheral blood mononuclear cells

## Conflict of Interest Disclosures

The authors declare that they have no competing interests.

## Authors' contributions

Study design (EGN, JJ, KAS, NLG, SKG, WON), CardioGene clinical enrollment and blood and data collection (DRH, KAS, KON, LM, RDS, SKG, WON), deCODE clinical enrollment and data collection (AH, KA, GT, TG), RNA expression data generation (EMB, KON, SKG), Data analysis (JJ, SKG), Manuscript preparation (AH, DRH, EGN, JJ, SKG, WON).

All authors read and approved the final manuscript.

## Pre-publication history

The pre-publication history for this paper can be accessed here:

http://www.biomedcentral.com/1755-8794/4/20/prepub

## Supplementary Material

Additional file 1 **Supplementary Table 1**. Initial 46 probesets identified in the timecourse analysis of the CardioGene samples. Click here for file

Additional file 2 **Supplementary Table 2**. Gene ontology annotation of the final 32 genes identified and validated. Click here for file

Additional file 3 **Supporting Materials**. Statistical methods and lists of probe IDs discovered and replicated. Click here for file
