# Supplementary material for: Time course analysis of gene expression identifies multiple genes with differential expression in patients with in-stent restenosis
Source: BMC Med Genomics. 2011 Feb 28;4:20. doi: 10.1186/1755-8794-4-20 (PMC3053213; doi:10.1186/1755-8794-4-20)
Supplement: Additional file 3 — Supporting Materials. Statistical methods and lists of probe IDs discovered and replicated. [file 1755-8794-4-20-S3.DOCX]

**Additional File 3**

**Supporting materials**

Statistical Model

For each gene, let $y_{ij}=y_{i}(t_{ij})$ be the expression level of individual $i$ measured at time$t_{ij}$, where $t_{ij}$ is the time when the $j$th measurement of the $i$th individual is obtained. Using the varying intercept model^14^, $y_{ij}$ is modeled by

$y_{ij}=\beta(t_{ij})+\varepsilon_{i}(t_{ij})$,

where $\beta(t)$ is the population average expression curve and $\varepsilon_{i}(t)$ is random error of individual $i$from this curve at time $t$. Here $\beta(t)$ is approximated by a basis function expansion by

$\beta(t)=\sum_{s=0}^{K} \gamma_{s}B_{s}(t)$,

where$B_{s}$ for $s=0,...,K$ is a set of B-spline basis, and the least square basis estimator of $\beta(t)$ given by

$$\hat{\beta}(t)=\sum_{s=0}^{K} \hat{\gamma}_{s}B_{s}(t)$$

can be obtained by minimizing the sum of squares between $\hat{\beta}(t_{ij})$ and $y_{ij}$^14^.

To test whether there is a significant difference between the curves of ISR and non-ISR patients, a goodness-of-fit test based on the comparison between the residual sum of squares under the null and alternative hypothesis was considered. Under the null hypothesis, a single curve was fit using the combined ISR and non-ISR patients, and the residual sum of squares from the least square fit, $RSS_{0}$, is obtained. Two separate curves were fit for the ISR and non-ISR patients under the alternative hypothesis and similarly the residual sum of squares, $RSS_{1}$, is obtained. The test statistic was then calculated as

$T=(RSS_{0}-RSS_{1})/RSS_{1}$.

The null distribution of this test statistic is assessed based on 10 million bootstrap samples obtained by sampling a number of subjects as described in Huang, Wu and Zhou^14^.

List of probes identified in the CardioGene discovery analysis:

201222_s_at

217814_at

203276_at

218047_at

208374_s_at

209207_s_at

202502_at

203996_s_at

212803_at

201181_at

217758_s_at

214773_x_at

213389_at

203044_at

200084_at

218092_s_at

208857_s_at

216304_x_at

209345_s_at

203994_s_at

212411_at

213654_at

212460_at

221423_s_at

222294_s_at

203371_s_at

200821_at

221504_s_at

210849_s_at

212513_s_at

201223_s_at

218646_at

203605_at

204829_s_at

217886_at

221613_s_at

202654_x_at

218341_at

212016_s_at

220131_at

201807_at

221123_x_at

205126_at

215203_at

205202_at

201351_s_at

List of probes validated in the analysis of decode samples:

210849_s_at

209207_s_at

208857_s_at

208374_s_at

212411_at

209345_s_at

205202_at

203994_s_at

217886_at

220131_at

217758_s_at

217814_at

212513_s_at

202654_x_at

202502_at

216304_x_at

200821_at

201807_at

218092_s_at

201222_s_at

201351_s_at

218646_at

214773_x_at

203996_s_at

203371_s_at

203605_at

218047_at

212460_at

221123_x_at

212803_at

222294_s_at

205126_at

221504_s_at

201223_s_at

203276_at

204829_s_at
